# Identification of SEPP1 polymorphisms is not a genetic risk factor for preeclampsia in Chinese Han women: A clinical trial and experimental study: Erratum

**DOI:** 10.1097/MD.0000000000009474

**Published:** 2017-12-22

In the article, “Identification of SEPP1 polymorphisms is not a genetic risk factor for preeclampsia in Chinese Han women: A clinical trial and experimental study”^[1]^, which appeared in Volume 96, Issue 28 of Medicine, the authors would like to add Dr. Jiangli Wang as a corresponding author.

Jiangli Wang, Prenatal Diagnosis Center, the Affiliated Hospital of Qingdao University, Qingdao, China (email: wangjingliya@126.com)
